# Prevalence and Clinical Implications of Incidentally Detected Parotid Lesions as Blind Spot on Brain MRI: A Single-Center Experience

**DOI:** 10.3390/medicina57080836

**Published:** 2021-08-18

**Authors:** In-Chul Nam, Hye-Jin Baek, Kyeong-Hwa Ryu, Jin-Il Moon, Eun Cho, Hyo-Jung An, Seokho Yoon, Jiyeon Baik

**Affiliations:** 1 Department of Radiology, Gyeongsang National University School of Medicine and Gyeongsang National University Changwon Hospital, Changwon 51472, Korea; sky_hall@naver.com (I.-C.N.); ryukh0329@gmail.com (K.-H.R.); drlotus@naver.com (J.-I.M.); sgeisilver@naver.com (E.C.); 2 Institute of Health Sciences, Gyeongsang National University School of Medicine, Jinju 52727, Korea; 3 Department of Pathology, Gyeongsang National University School of Medicine and Gyeongsang National University Changwon Hospital, Changwon 51472, Korea; ariel2020@naver.com; 4 Department of Nuclear Medicine and Molecular Imaging, Gyeongsang National University School of Medicine and Gyeongsang National University Changwon Hospital, Changwon 51472, Korea; yoon.seokho@gmail.com; 5 Department of Radiology, Haeundae Paik Hospital, Inje University College of Medicine, Busan 48108, Korea; jbaik6@gmail.com

**Keywords:** incidental parotid lesions, parotid gland, parotid neoplasm, brain, magnetic resonance imaging

## Abstract

*Background and objective*: This study was conducted to assess the prevalence and clinical implications of parotid lesions detected incidentally during brain magnetic resonance imaging (MRI) examination. *Materials and Methods*: Between February 2016 and February 2021, we identified 86 lesions in the brain MRI reports of 84 patients that contained the words “parotid gland” or “PG”. Of these, we finally included 49 lesions involving 45 patients following histopathological confirmation. *Results*: Based on the laboratory, radiological or histopathological findings, the prevalence of incidental parotid lesions was low (1.2%). Among the 45 study patients, 41 (91.1%) had unilateral lesions, and the majority of the lesions were located in the superficial lobe (40/49, 81.6%). The mean size of the parotid lesions was 1.3 cm ± 0.4 cm (range, 0.5 cm–2.8 cm). Of these, 46 parotid lesions (93.9%) were benign, whereas the remaining three lesions were malignant (6.1%). *Conclusions*: Despite the low prevalence and incidence of malignancy associated with incidental parotid lesions detected on brain MRI, the clinical implications are potentially significant. Therefore, clinical awareness and appropriate imaging work-up of these lesions are important for accurate diagnosis and timely management.

## 1. Introduction

Radiologists frequently encounter incidental findings in all imaging modalities during routine clinical practice [1,2,3]. Over the years, the prevalence of incidental findings has increased because of the increased demand for accurate diagnostic imaging, as well as rapid technical improvements and diagnostic quality of imaging modalities in the radiology department [4,5,6]. In particular, the number of brain magnetic resonance imagings (MRIs) for evaluating various intracranial diseases has dramatically increased. Thus, radiologists may increasingly encounter asymptomatic incidental parotid lesions within the scan range. However, the management of incidental lesions detected on brain MRI is limited by a lack of knowledge regarding their clinical significance, partial visualization of lesions, and non-availability of dedicated additional sequences for the incidental lesions [7].

The parotid gland is the largest salivary gland and is located in the parotid space near the ear [8]. A wide variety of lesions are associated with the parotid gland, such as congenital anomalies, inflammatory or infectious processes, and benign or malignant neoplasms [9]. Although the majority of the parotid lesions are benign [10], incidental parotid lesions may require additional diagnostic work-ups to exclude malignancies and facilitate appropriate management. A few imaging studies have reported the presence of unexpected asymptomatic parotid lesions; however, these studies mainly focused on parotid lesions detected via 18-F fluorodeoxyglucose (FDG) positron emission tomography (PET) or PET–computed tomography (CT) [11,12,13,14,15]. In contrast, the clinical significance of incidental parotid lesions detected via other more commonly utilized imaging modalities remains unclear [16]. Furthermore, the frequency of the incidental findings in previous studies has varied according to the imaging modality used and the area of interest investigated [11,12,13,14,15,16]. Therefore, the purpose of our retrospective study was to assess the prevalence and clinical implications of incidental parotid lesions as potential blind spots detected during brain MRI examination.

## 2. Materials and Methods

### 2.1. Patients

The Institutional Review Board of Gyeongsang National University Changwon Hospital approved the study (No.: GNUCH 2021-03-012) on 25 March 2021. However, no patient approval or informed consent was required due to the retrospective nature of the study.

**Figure 1:**
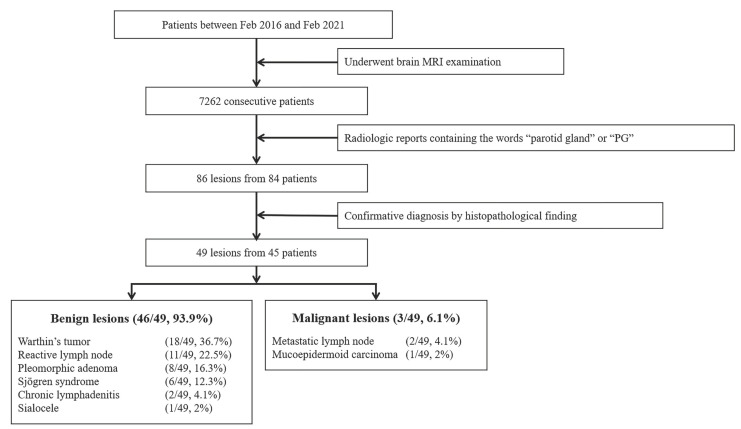
Flow chart. Magnetic resonance imaging (MRI).

We retrospectively reviewed our institutional database and identified 7262 patients who underwent diagnostic brain MRI examinations from 1 February 2016 to 1 February 2021. Of these, we analyzed 86 lesions in the radiologic reports of brain MRI belonging to 84 patients that contained the words “parotid gland” or “PG”. Using the electronic medical record, as well as the picture archiving and communication system (PACS), we included 49 lesions in 45 patients with histopathologically confirmed diagnoses (Figure 1). Forty-three of the final 45 patients underwent contrast-enhanced brain MRI, whereas 2 patients underwent unenhanced brain MRI.

The study patients consisted of 24 (53.3%) men and 21 (46.7%) women (age range, 18 –87 years; mean age, 51.7 ± 12.3 years). Of the 45 patients, 35 patients (77.8%) had no previous medical history, whereas 10 patients (22.2%) had a history of malignancy, which included lung cancer in four patients, nasopharyngeal cancer in two patients, tonsil cancer in two patients, colon cancer in one patient, and breast cancer in one patient.

### 2.2. Brain MRI Parameters

MRI was performed using two 3T MR scanners. Our conventional brain MRI protocol included the following sequences: axial T2-weighted fast spin-echo imaging with the Dixon technique, axial T1-weighted fluid attenuation inversion recovery imaging (T1-FLAIR), axial T2-weighted fluid attenuation inversion recovery imaging (T2-FLAIR), diffusion-weighted imaging, and susceptibility-weighted imaging with or without contrast-enhanced 3D T1-weighted gradient-echo imaging. The examination of 25 patients was performed on a 3T MR scanner with a 48-channel head coil (Signa^™^ Architect; GE Healthcare, Waukesha, WI, USA), whereas the other 20 patients underwent brain MRI using another 3T MR scanner with a 32-channel head coil (Ingenia 3.0 CX; Philips Healthcare, Best, The Netherlands).

### 2.3. Assessment of Incidental Parotid Lesions: Image Analyses

The brain MRIs were reviewed by an attending neuroradiologist (H.J.B) with 11 years of experience evaluating incidental parotid lesions within the scan range. The reader assessed the digital PACS for the lesion size, location (superficial lobe/deep lobe), bilaterality, multiplicity, contrast enhancement, and additional imaging studies (i.e., neck ultrasound (US), neck CT, PET–CT, or salivary gland scintigraphy).

A clinical lecturer with one year of experience in interventional radiology (I.C.N) reviewed and categorized the parotid lesions according to the final diagnosis based on clinical and laboratory findings as well as histopathological analyses using electronic medical records.

### 2.4. Statistical Analysis

The prevalence of incidental parotid lesions and individual disease entities was analyzed statistically. Continuous variables were expressed as the mean ± standard deviation (SD).

## 3. Results

Among the total 86 parotid lesions detected on brain MRI incidentally, 49 lesions were confirmed via US-guided fine-needle aspiration biopsy (31/49, 63.3%) or US-guided core needle biopsy (18/49, 36.7%) in 45 patients, including four patients who underwent US-guided core needle biopsy for bilateral parotid lesions. In the study, 28 patients (62.2%) underwent superficial parotidectomies.

Of the 49 lesions, 47 (95.9%) were only detected on contrast-enhanced 3D T1-weighted images. All 49 lesions were evaluated in subsequent imaging studies, including neck US (49/49, 100%), neck CT (18/49, 36.7%), PET–CT (9/49, 18.4%), and salivary gland scintigraphy (6/49, 12.2%).

Of the 45 patients, only four patients (8.9%) carried bilateral parotid lesions. However, the lesions of only two of these four patients were identified on brain MRI because of the limited scan range and head tilting during the scan. Bilateral parotid lesions were confirmed in the other two patients via other imaging studies (US and salivary scintigraphy).

In addition, the majority of the parotid lesions (40/49, 81.6%) were found in the superficial lobe. Five of the remaining nine lesions (5/49, 10.2%) were located in the deep lobe, whereas four lesions (4/49, 8.2%) were identified in both superficial and deep lobes. The mean size of the incidental parotid lesions was 1.3 ± 0.4 cm with a range of 0.5–2.8 cm; 17 lesions were less than 1 cm (34.7%), 25 lesions were between 1 and 2 cm (51%), and 7 lesions (14.3%) were larger than 2 cm.

**Figure 2:**
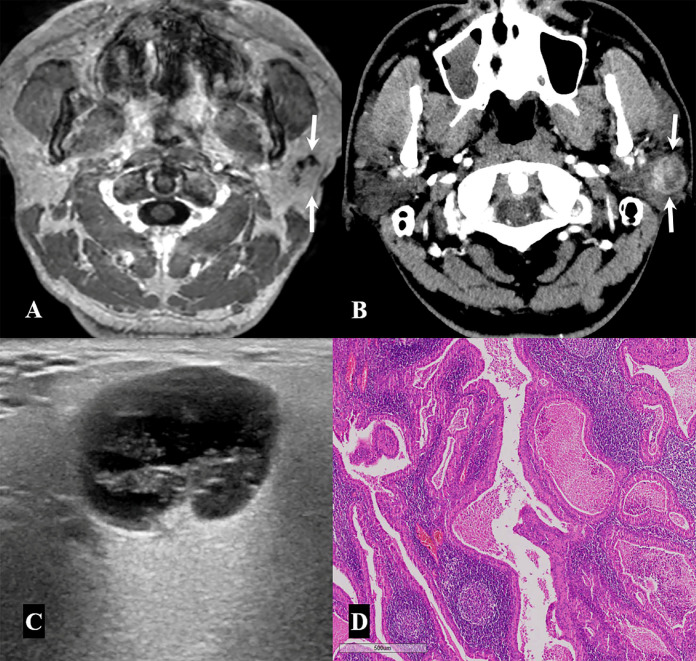
A 48-year-old man with a Warthin tumor. (**A,B**) Contrast-enhanced axial T1-weighted image (**A**) and enhanced axial neck computed tomography (CT) image (**B**) show a well-circumscribed, oval, heterogeneously enhancing mass with intratumoral cystic components in the left parotid gland. (**C**) The mass demonstrates mixed cystic and solid features with posterior acoustic enhancement on neck ultrasonography. (**D**) Histopathological examination of a specimen from the superficial parotidectomy shows double layers of oncocytic tumor cells in the background of dense lymphoid stroma and many germinal centers, suggesting a Warthin tumor (×40 magnification; hematoxylin and eosin stain).

**Figure 3:**
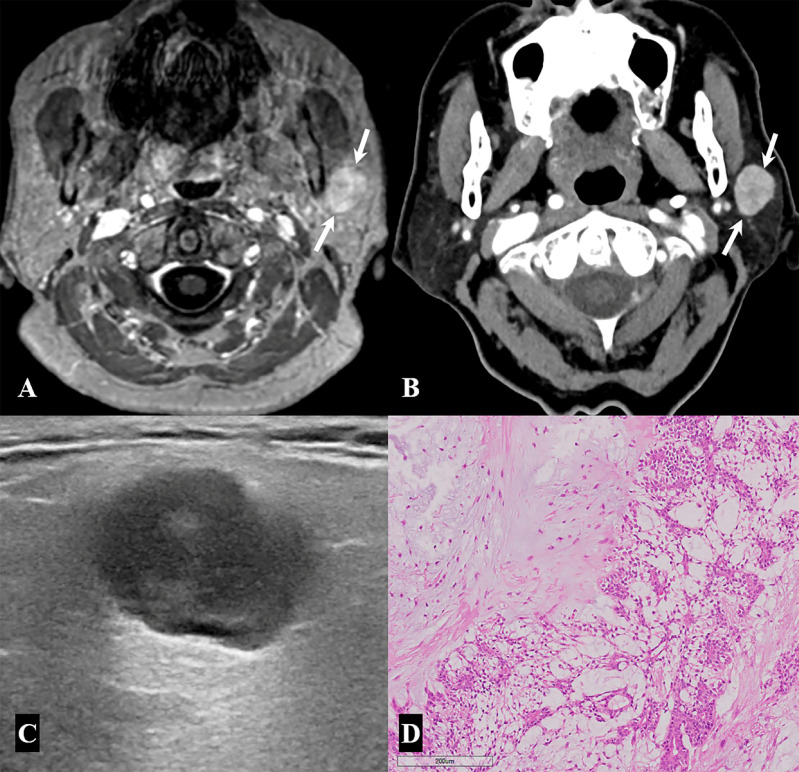
A 76-year-old woman with pleomorphic adenoma. (**A,B**) Contrast-enhanced axial T1-weighted image (**A**) and delayed enhanced axial neck CT image (**B**) show a well-circumscribed, oval, homogeneously enhancing mass in the left parotid gland, superoanterior portion. (**C**) The mass demonstrates marked hypoechogenicity with a lobulated margin and posterior acoustic enhancement on neck ultrasonography. (**D**) Histopathological examination of a specimen from the superficial parotidectomy shows epithelial and myoepithelial cells forming the inner and outer layers of cysts and tubules scattered in the background of myxochondroid stroma, suggesting pleomorphic adenoma (×100 magnification; hematoxylin and eosin stain).

**Figure 4:**
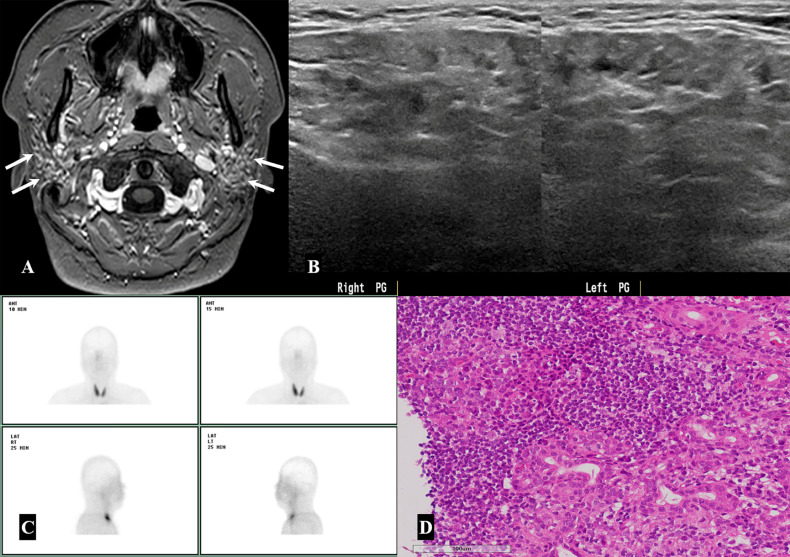
A 46-year-old woman with Sjögren’s syndrome. (**A,B**) On the contrast-enhanced axial T1-weighted image, there are multiple tiny enhancing granulonodular foci in both parotid glands with inhomogeneous parenchymal signal intensities. (**B**) On neck ultrasonography, the bilateral parotid glands show multiple tiny hypoechoic nodules with coarse parenchymal echotexture and decreased parenchymal echogenicity. (**C**) Salivary gland scintigraphy demonstrates severely decreased uptake in the bilateral parotid and submandibular glands. (**D**) Histopathological examination of ultrasound-guided core needle biopsy sample shows extensive lymphoid infiltration and interstitial fibrosis with a germinal center, suggestive of Sjögren’s syndrome (×200 magnification; hematoxylin and eosin stain).

The final diagnosis based on histopathological reports, as well as clinical and laboratory findings, indicated that 46 parotid lesions (93.9%) were benign and only three (6.1%) were malignant (Table 1). Of the 46 benign parotid lesions, 40 (40/46, 87%) were focal lesions manifesting as nodules or masses, whereas the remaining 6 (13%) were diffuse glandular lesions. The most common diagnosis of the 40 focal lesions was Warthin tumor (18/49, 36.7%), followed by reactive lymph node (11/49, 22.5%), and pleomorphic adenoma (8/49, 16.3%) (Figure 2 and Figure 3). However, only one of the patients with a Warthin tumor showed bilaterality. The six diffuse glandular lesions with bilateral involvement in three patients were associated with Sjögren’s syndrome characterized by decreased salivary excretion in the bilateral parotid and submandibular glands in salivary gland scintigraphy, as well as positive test results for anti-SS-A or anti-SS-B antibodies (Figure 4).

**Figure 5:**
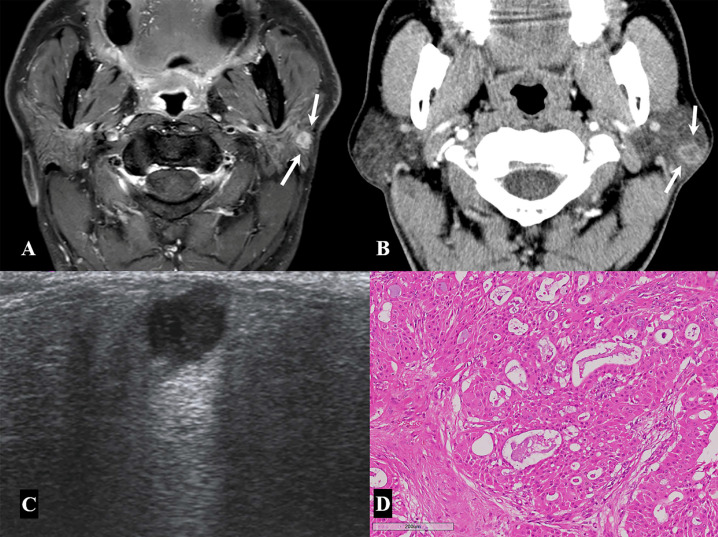
A 58-year-old man with low-grade mucoepidermoid carcinoma. (**A,B**) Contrast-enhanced axial T1-weighted image (**A**) and delayed enhanced axial neck CT image (**B**) show a small enhancing nodular lesion with a central non-enhancing portion in the left parotid gland. (**C**) On neck ultrasonography, there is a markedly hypoechoic nodule with a focal spiculated margin and posterior acoustic enhancement in the superficial lobe of the left parotid gland. (**D**) Histopathological examination of a specimen from the superficial parotidectomy shows sheets of mucous, squamous, and intermediate cells with bland-looking nuclei forming glandular spaces, suggestive of low-grade mucoepidermoid carcinoma (×100 magnification; hematoxylin and eosin stain).

The three malignant parotid lesions comprised metastatic lymph nodes (2/49, 4.1%) and mucoepidermoid carcinoma (1/49, 2%) (Figure 5).

## 4. Discussion

The increased use of brain MRI for evaluating various intracranial abnormalities has increased the frequency of encountering incidental findings such as parotid lesions within the radiological scan range. Unfortunately, these incidental findings can be a diagnostic challenge for radiologists because of the limitations associated with the handling of incidental lesions detected on brain MRI: (1) lack of adequate knowledge regarding their clinical significance; (2) partial visualization of the majority of incidental lesions; (3) non-availability of dedicated additional MRI sequences [7].

The reported prevalence of incidental parotid lesions on cross-sectional imaging ranges between 0.3% and 0.45%. Nearly 32% of these lesions are malignant on PET imaging. However, the prevalence of incidental parotid lesions based on CT and MRI is still unknown. To the best of our knowledge, only a single retrospective study reviewed incidental parotid lesions on CT, MRI, and PET imaging [16]. Therefore, we retrospectively assessed the prevalence and clinical implications of incidental parotid lesions as blind spots detected only on brain MRI.

In this study, the majority of the parotid lesions (95.9%) were visualized only in contrast-enhanced 3D T1-weighted images, probably because this sequence has a wider scan range than the other sequences in our routine brain MRI protocol. We also found that the prevalence of incidental parotid lesions on brain MRIs was 1.2% (84/7262), which was three- to four-fold higher than the results in previous studies using PET [12,14,15]. The reason for this discrepancy is unclear. However, it appears to be related to the intrinsic differences between the two imaging modalities of brain MRI and PET or PET–CT.

Parotid lesions without FDG uptake are undetectable in PET or PET–CT, which are functional imaging techniques used to detect tissue metabolism representing the degree of glucose utilization using a glucose analog as an indicator [17]. By contrast, parotid lesions are more easily detected using brain MRI if the lesion is within the scan range because MRI is an anatomically oriented cross-sectional imaging modality. In addition, PET and PET–CT used to detect these lesions are limited by poor resolution and the partial volume effect. Furthermore, PET or PET–CT is usually performed during the initial staging work-up or follow-up of cancer patients [12]. Therefore, the clinical indications are limited compared to brain MRI.

In the current study, the average size of the lesions was 1.3 cm, similar to the results of a previous study, suggesting that the small size of parotid lesions is a possible cause of asymptomatic conditions [16]. In addition, most of the parotid lesions (81.6%) were located in the superficial lobe, which is consistent with the results of previous studies that described the imaging characteristics of parotid lesions [18,19,20]. However, only four patients had bilateral parotid lesions, which is inconsistent with previous studies, which reported up to 25% [16,18,19,20]. The reason for this discrepancy is unclear, but it is probably related to the differences in the number of enrolled study patients and the histopathology of the lesions. We also found that the lesions were undetectable on brain MRI due to the limited scan range and inadequate scan position, such as head tilting, limiting the evaluation of bilaterality or multiplicity. Therefore, a subsequent imaging work-up is needed to determine the clinical significance of incidental parotid lesions.

Our results suggest that the majority of the parotid lesions (93.9%) were benign, and the most common histopathology was Warthin tumors (36.7%), followed by reactive lymph nodes (22.5%) and pleomorphic adenomas (16.3%). These findings were inconsistent with previous studies regarding symptomatic parotid tumors, which reported pleomorphic adenoma as the most common tumor in the parotid gland [21,22]. However, they are consistent with recent studies suggesting that Warthin tumors were the most frequent benign lesion in asymptomatic parotid lesions [16,23]. Al-Balas et al. [16] reported that more than 50% of the lesions were Warthin tumors, followed by pleomorphic tumors. Bothe et al. [23] found a similar preponderance of Warthin tumors, constituting 83% of the asymptomatic parotid lesions. Additionally, the difference in the frequency of Warthin tumors between this study and previous studies may be related to the differences in the number of enrolled study patients. However, the findings of our study and previous studies cannot reflect data in real-world practice because of the selection bias related to the clinical characteristics of the study patients (i.e., age, sex or smoking status) and the use of PET or PET–CT. In particular, it is well known that Warthin tumors often reveal increased FDG uptake that is often equivalent to the uptake level by malignant tumors, and the previous studies using PET or PET–CT demonstrated a higher prevalence of Warthin tumors as incidentalomas in the parotid gland [24,25,26,27].

We found that the overall incidence of parotid malignancy was 6.1%, including metastatic lymph nodes (4.1%) and primary salivary gland tumors (2%). These results are consistent with previous studies that reported an incidence in the range of 3.1–7.9% for asymptomatic or incidentally detected parotid lesions [16,28,29]. We also found that the risk of malignancy in incidental parotid lesions was substantially lower in patients without a history of malignancy (1/35, 2.9%) than in patients with underlying malignancy (2/10, 20%).

The study limitations should be considered when interpreting the results. First, this study was retrospectively designed, which suggests selection bias. We included study patients by reviewing the radiologic reports. Therefore, it is difficult to completely exclude the possibility of parotid lesions on brain MRI missed by radiologists during the initial imaging assessment. Considering this issue, the actual prevalence of incidental parotid lesions on brain MRI might be higher than the results of the current study. Second, the sample size was small. Third, routine brain MRI and MR protocols vary according to the institution, and these factors can affect the prevalence of incidental parotid lesions.

Despite these limitations, our study may be useful in understanding incidental parotid lesions detected on brain MRI. Additional imaging studies and histopathological analyses are needed to demonstrate their prevalence and the associated risk of malignancy. Therefore, further studies with a larger sample size and different brain MRI protocols and MR systems can facilitate the validation of our findings and propose a lesion-specific diagnostic workflow using rational and cost-effective strategies for the management of incidentally detected parotid lesions on brain MRI.

## 5. Conclusions

In conclusion, we found a low prevalence of incidental parotid lesions on brain MRI. These lesions also showed a low risk of malignancy. However, the clinical significance of these lesions should not be overlooked in daily clinical practice, and appropriate imaging work-up is invaluable in evaluating incidental parotid lesions and avoiding unnecessary biopsies. Therefore, clinical awareness and meticulous image inspection are important for accurate diagnosis and timely management of incidental parotid lesions detected on brain MRI. In addition, our observations underscore the need for a subsequent study with a larger patient population to investigate the clinical implications and management of the incidental findings beyond the intracranial space based on ethical concerns and healthcare policy.

## Figures and Tables

**Table 1:** Final diagnosis of 47 incidental parotid lesions based on histopathologic results.

Histopathology (*n* = 49)	Number of Lesions (%)
Benign Lesion (*n* = 46)	
Warthin tumor	18/49 (36.7%)
Reactive lymph node	11/49 (22.5%)
Pleomorphic adenoma	8/49 (16.3%)
Sjögren’s syndrome	6/49 (12.3%)
Chronic lymphadenitis	2/49 (4.1%)
Sialocele	1/49 (2 %)
Malignant lesion (*n* = 3)	
Metastatic lymph node	2/49 (4.1%)
Mucoepidermoid carcinoma	1/49 (2 %)

## Data Availability

The anonymized data that support the results of this study are available and shared on reasonable request by any qualified investigator.
